# A Biological Insight into the Susceptibility to Influenza Infection in Junior Rats by Comprehensive Analysis of lncRNA Profiles

**DOI:** 10.1155/2021/8112783

**Published:** 2021-08-17

**Authors:** Fen-Sheng Qiu, Hao-Kun Chen, Hua-Zhong Ying, Wen-Ying Yu, Mei-Ying Guo, Wen-Wei Zhou

**Affiliations:** Zhejiang Provincial Laboratory of Experimental Animal's & Nonclinical Laboratory Studies, Hangzhou Medical College, Hangzhou 310013, China

## Abstract

Long noncoding RNAs (lncRNAs) have been reported to participate in regulating many biological processes, including immune response to influenza A virus (IAV). However, the association between lncRNA expression profiles and influenza infection susceptibility has not been well elucidated. Here, we analyzed the expression profiles of lncRNAs, miRNAs, and mRNAs among IAV-infected adult rat (IAR), normal adult rat (AR), IAV-infected junior rat (IJR), and normal junior rat (JR) by RNA sequencing. Compared with differently expressed lncRNAs (DElncRNAs) between AR and IAR, 24 specific DElncRNAs were found between IJR and JR. Then, based on the fold changes and *P* value, the top 5 DElncRNAs, including 3 upregulated and 2 downregulated lncRNAs, were chosen to establish a ceRNA network for further disclosing their regulatory mechanisms. To visualize the differentially expressed genes in the ceRNA network, GO and KEGG pathway analysis was performed to further explore their roles in influenza infection of junior rats. The results showed that the downregulated DElncRNA-target genes were mostly enriched in the IL-17 signaling pathway. It indicated that the downregulated lncRNAs conferred the susceptibility of junior rats to IAV via mediating the IL-17 signaling pathway.

## 1. Introduction

Influenza is an infectious respiratory disease mainly caused by influenza viruses [1]. Against the background of the continuous COVID-19 coronavirus pandemic, influenza virus is still a serious threat to public health with not only morbidity from 25% to 90% but also the mortality rate that is fluctuated from 1% to 3% and more than 500,000 people all over the world die from influenza every year [2, 3]. Notably, approximately 870,000 children in preschool are annually hospitalized worldwide due to influenza [4]. Though infectious respiratory disease can occur in all ages, children are more susceptible to influenza infection and have more severe symptoms because of the imperfect immune system [5]. Compared with other age groups, rates of influenza infection are usually the highest in children, especially in children with underlying chronic medical conditions [6]. Moreover, it is reported that host genetic factors play an important role on child susceptibility to influenza infection [7]. However, the molecular mechanisms underlying the influenza-associated genes and progression of influenza remain largely ambiguous.

Long noncoding RNAs (lncRNAs) are defined as a series of transcript RNAs which are more than 200 nucleotides with limited or no protein-coding capacity [8–10]. Accumulating evidences indicate that they perform various functions as regulatory RNAs in diverse biological processes [11–14]. Recently, lncRNAs have been reported to act as prominent regulators of virus-host interactions [15–19]. Although host lncRNAs have been proved to involve in regulating innate immunity against viral infection and replication, the relationship between lncRNA profiles and child susceptibility has been unknown [20].

In this study, we performed RNA sequencing (RNA-seq) in the rat samples to investigate the host immune response to influenza A virus (IAV). Based on RNA-seq data, the specific differentially expressed lncRNAs (DElncRNAs) were identified to construct a ceRNA regulatory network. Furthermore, the molecular mechanisms of the differentially expressed genes were investigated by bioinformatic analysis. Therefore, this study explored the DElncRNA profiles as the potential targets against IAV infection and uncovered a novel regulatory mechanism, which would provide a new insight into the susceptibility factors for IAV infection in children.

## 2. Materials and Methods

### 2.1. IAV-Infected Rat Model

Male SD rats (aged 3 and 6 weeks old) were purchased from Zhejiang experimental animal center (Zhejiang, China) and bred under specific pathogen-free conditions. The influenza A virus (H1N1 subtype) was obtained from the Zhejiang Provincial center for disease control and prevention (Zhejiang, China). The infection was induced under sevoflurane by nasal inoculation of IAV, which had been challenged for 5 times in mice with tissue culture infective dose (TCID_50_) of 10^3.5^ [21]. After intranasally infected with IAV, the rats were randomly divided into four groups (namely, IAV-infected adult rat (IAR), normal adult rat (AR), IAV-infected junior rat (IJR), and normal junior rat (JR)). Each group had 5 rats, and the rats were sacrificed at day 3 postinfection. The experiments were performed following protocols approved by the animal ethics committee, Zhejiang experimental animal center, and implemented in accordance with the local guide for the care and use of laboratory animals.

### 2.2. Histopathology

The whole lungs of each group were stripped away and fixed with 10% neutral buffered formalin after perfused with 5 ml of PBS. The fixed tissues were embedded in paraffin, sectioned into 5 *μ*m sections, deparaffinized, rehydrated, and then stained with hematoxylin and eosin. Infections in the lungs were assessed by the level of the formation of lymphoid aggregates and leucocyte infiltration of the airway space, combined with the degree of perivascular lymphocytic aggregation within the sections [22, 23].

### 2.3. RNA Sample Collection and Library Preparation

Three micrograms of total RNA is from each sample of IAR, AR, IJR, and JR as the initial amount to establish four lncRNA libraries. Firstly, an Ribo-zero™ GoldKits (RiBOBiTech, Guangzhou, China) was used to remove ribosomal RNA (rRNA). Secondly, different index tags were chosen to establish lncRNA libraries according to the instructions of the NEB Next Ultra Directional RNA LibraryPrep Kit for Illumina (NEB, Ispawich, USA). Finally, the established libraries were used for Illumina sequencing.

### 2.4. RNA-seq Data Acquisition and Quality Control

After RNA-seq, a good deal of sequencing raw data was obtained from 4 independent samples. To ensure the quality of informatic analysis data, we filtered the raw reads to acquire high-quality clean reads by avoiding adapter-polluted reads, low-quality reads, Ns reads, and rRNA mapping reads and then performed further bioinformatic analysis. Bioinformatic analysis was based on the clean reads.

### 2.5. Differential Expression Analysis of lncRNAs

The expression values of lncRNAs in each sample were normalized using the Fragments per Kilobase per Million Mapped Fragments (FPKM). FPKM was defined per million sequence number as the number of lncRNA expression, of which the total number of aligned reads were used by normalized expression values. The data of the lncRNAs from 4 samples was separately recombined, to make a comparison using DEGseq software. The DElncRNAs were filtrated with ∣log2Ratio | ≥1.5 and *q* ≤ 0.05 as the screening conditions to obtain the up- and downregulated genes.

### 2.6. Construction of ceRNA Regulatory Network

The interactions between DElncRNAs and miRNA were predicted using miRanda, PITA, and TargetScan. The lncRNA-miRNA interaction pairs were recognized as targeted relationship when successfully predicted in two websites at least. In addition, miRNA-mRNA interaction pairs predicted by the above method were also selected. Next, the lncRNA-miRNA-mRNA ceRNA regulatory network was established by Cytoscape software (Version 3.8.2) [24].

### 2.7. Functional Enrichment Analysis of Differentially Expressed lncRNAs

To illustrate gene ontology or molecular pathway enrichment, the WEB-based Gene SeT AnaLysis Toolkit (WebGestalt) was used to perform GO functional annotation, including biological process (BP), cellular component (CC), and molecular function (MF), and to analyze KEGG pathway enrichment for the significant DElncRNA target genes [25].

### 2.8. Statistical Analysis

Normalization FPKM analysis was used to control the quality of the sequence data of lncRNAs, miRNAs, and mRNAs in our study. Statistical comparisons of the data were analyzed by SPSS software (SPSS 26, Chicago, IL, USA). Multiple comparisons among all groups were performed by one-way analysis of variance (ANOVA). Meanwhile, comparisons between two groups were performed by Student's test. *P* < 0.05 was considered as a statistically significant difference.

## 3. Results

### 3.1. Lung Histopathology

**Figure 1:**
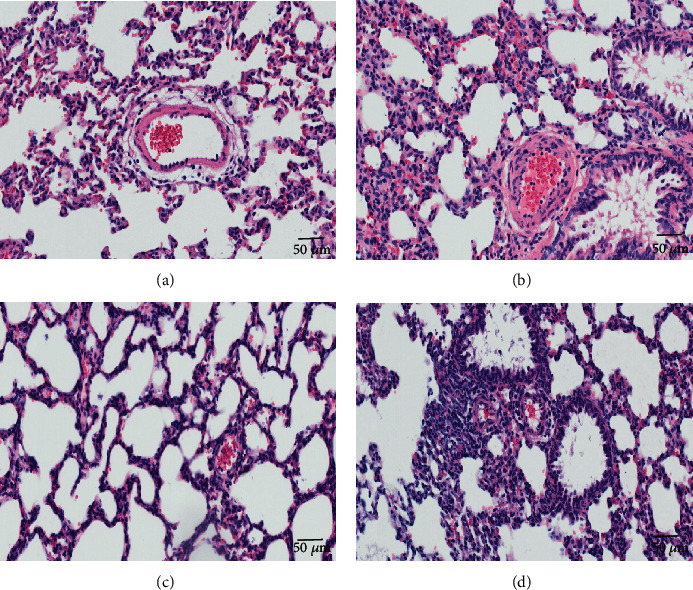
Histopathological analysis for lung tissues at day 3 postinfection. Representative HE-stained sections of lung tissues from (a) normal adult rat (AR), (b) influenza-infected adult rat (IAR), (c) normal junior rat (JR), and (d) influenza-infected junior rat (IJR). Scale bar = 50 *μ*m for all images.

The results of hematoxylin and eosin staining clearly showed that compared with IAV-uninfected (AR and JR) groups, the lung tissue sections of IAV-infected (IAR and IJR) groups had higher levels of leucocyte infiltration and platelet aggregation in the airway and perivascular spaces (Figure 1). However, those features were hardly found in uninfected groups. It suggested the serious lung injury and inflammation induced by IAV.

### 3.2. Sequencing Data Filtering and Alignment Analysis

After RNA-seq, plentiful raw data were got from 4 samples (AR, IAR, JR, and IJR). According to the manufacturer protocols, the raw data were filtered to obtain high-quality reads and sequences for further bioinformatic analysis. The total numbers of raw reads, clean reads, and clean bases of all samples are shown in Table 1. To identify lncRNA sequences, we aligned the filtered data using HiSAT2 and the mapping rates were more than 95% of total reads, which indicated not only a high utilization of the sequencing reads but also reliable results in the subsequent analysis.

### 3.3. Identification of Differentially Expressed lncRNAs

**Figure 2:**
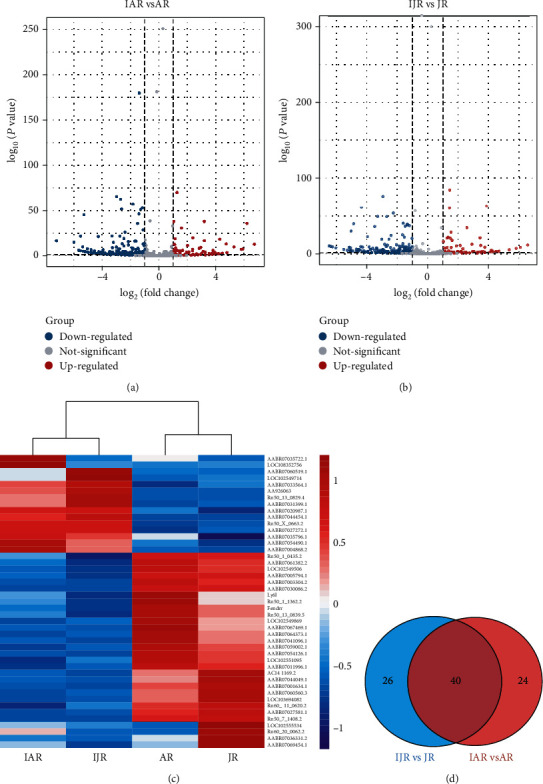
Identification of differentially expressed lncRNAs. The volcano plot showing the differentially expressed lncRNAs compared IAR with AR (a) and compared IJR with JR (b). (c) All the expression level of the differentially expressed lncRNAs in each sample was analyzed by the hierarchical cluster. (d) Venn diagram showing the overlap of DElncRNAs between the IAR vs. AR group and the IJR vs. JR group.

Based on the filtered data, compared with AR, 763 DelncRNAs were found in IAR, and similarly, 763 in IJR when compared with JR. In addition, ∣log2Ratio | ≥1.5 and *q* < 0.05 as the screening conditions were performed to further identify the DElncRNAs by using DEGseq. To visualize DElncRNAs between IAV-infected groups and normal groups in both adult and junior rats, the volcano plots and heatmaps were illustrated (Figure 2). Compared the expression profiles in IAR and AR samples, a total of 66 known DElncRNAs were selected for further analysis. Moreover, it was totally 64 known DElncRNAs, compared IJR with JR. To identify the unique known DElncRNAs between IJR and JR, we selected top 5 DElncRNAs filtered by fold changes and *P* value. The 5 specific DElncRNAs compared IJR with JR were selected as candidates for further bioinformatic analysis.

### 3.4. Targeted miRNA Analysis of Differentially Expressed lncRNAs

To determine the function of the 5 specific DElncRNAs, we predicted the targeted miRNAs of DElncRNAs by using three online websites: miRanda, PITA, and TargetScan. The lncRNA-miRNA interaction was recognized as targeted relationship when successfully predicted in two websites at least. Then, the targeted miRNAs were selected which were differentially expressed and negatively associated with the expressions of the lncRNAs. Finally, 25 lncRNA-miRNA pairs were identified and associated with the severity of IAV infection in junior rats after online prediction.

### 3.5. Identification of Potential ceRNA (lncRNA-miRNA-mRNA) Regulatory Network

**Figure 3:**
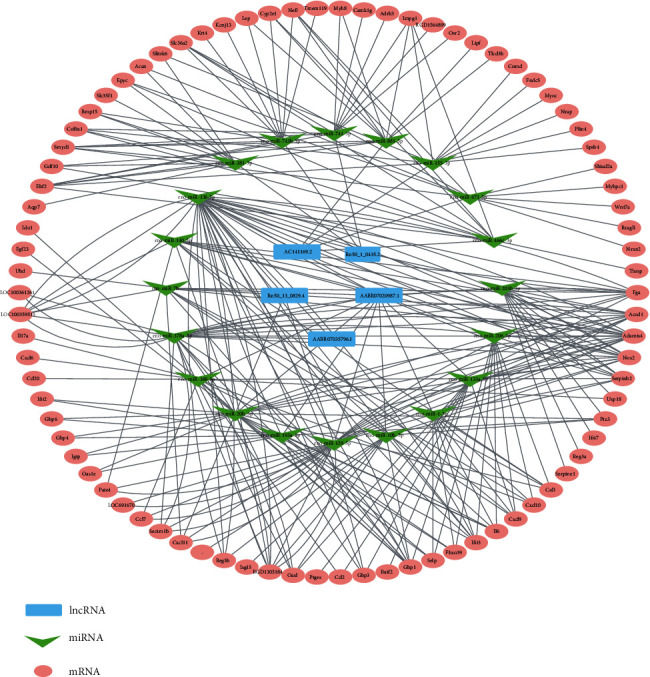
Identification of potential ceRNA (lncRNA-miRNA-mRNA) regulatory network. The lncRNA-miRNA-mRNA network of 3 upregulated DElncRNAs and 2 downregulated DElncRNAs was visualized in Cytoscape.

The miRNA-mRNA interaction pairs were also predicted by the same method according to the binding free energies and the binding mode. The targeted mRNAs were also negatively associated with the expression of miRNAs. As the translation of mRNAs might be regulated by lncRNAs via sponging miRNAs, a lncRNA-mRNA competing interaction pair would be considered if the mRNA and the lncRNA significantly shared common miRNAs. Thus, top 10 miRNA-mRNA interaction pairs of each DElncRNA-target miRNAs filtered by fold changes were selected in this study. Based on lncRNA-miRNA and miRNA-mRNA regulatory relationships, a competitive endogenous RNA (ceRNA) network of lncRNA-miRNA-mRNA was established to further explore the regulatory mechanisms of lncRNAs, including up- and downregulated lncRNAs (Figure 3). Then, we analyzed the ceRNA networks by using CytoHubba analysis and the hub miRNAs competitively bound by ceRNAs were presented, which included rno-miR-20-3p, rno-miR-136-5p, and rno-miR-378a-5p. Therefore, those results indicated that rno-miR-20-3p had been the potential acted as a novel prognostic indicator for IAV.

### 3.6. Functional Enrichment Analysis

**Figure 4:**
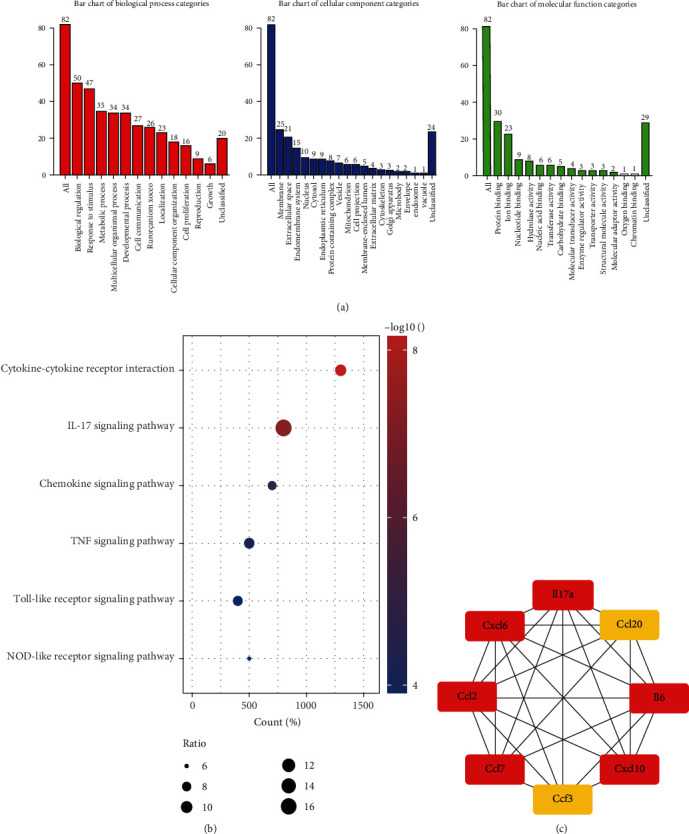
Functional enrichment analysis. (a) GO analysis for DEmRNAs in the ceRNA regulatory network, including the significant biological processes, molecular functions, and cellular components of DEmRNAs. (b) Bubble plots showing the crucial pathways for DEmRNAs by KEGG pathway analysis. (c) The IL-17 signaling pathway-related genes were evaluated by CytoHubba, with a high score shown in red and a low score shown in yellow.

To further investigate the roles of these DElncRNA-associated mRNA genes in ceRNA regulatory networks, GO and KEGG pathway analyses were performed by using WebGestalt database. The results showed that the obviously enriched BP included biological regulation, response to stimulus, and metabolic process. The CC contained membrane, extracellular space, and endomembrane system, whereas MF covered protein binding, ion binding, and nucleotide binding. On the other hand, these DElncRNA-associated mRNAs were annotated by KEGG pathway analysis. As shown in Figure 4, the function of these mRNAs was mainly involved in cytokine-cytokine receptor interaction, IL-17 signaling pathway, chemokine signaling pathway, TNF signaling pathway, and Toll-like receptor signaling pathway. Among these pathways, the IL-17 signaling pathway presented the largest gene hits, including IL-17A, CXCL16, CCL7, IL-6, CCL2, CXCL10, CCL20, and CSF3. Compared with CCL20 and CSF3, other proinflammatory cytokines and chemokines marked with red color in Figure 4(c) exerted high scores, indicating that they would play crucial roles in the susceptibility of junior rats to IAV.

## 4. Discussion

Influenza is an acute respiratory infection caused by influenza virus, which leads to respiratory symptoms, fever, and even a series of systemic symptoms in children [26]. Compared with adults, children as immunocompromised individuals are more susceptible to be infected with IAV [27, 28]. Therefore, it is vital to make a treatment plan to elucidate the susceptibility to children for clinical prevention and treatment of IAV infection. Numbers of evidences have illustrated that host lncRNAs act as either positive or negative regulators of the innate antiviral response, facilitating influenza virus replication [29–31]. In the study, we detected the gene expression profiles with the focus on lncRNAs in IAR, AR, IJR, and JR by using RNA-seq, having found plenty of differentially expressed lncRNAs. The results revealed that lncRNA AABR07020987.1, AABR07035796.1, and Rn50_13_0829.4 were significantly downregulated, while Rn50_1_0435.2 and AC141169.2 were significantly upregulated in IAV-infected junior rats, indicating that these DElncRNAs had potential to be novel biomarkers for evaluating the prognosis and diagnosis of IAV challenge. In addition, a ceRNA regulatory network was constructed to investigate the lncRNA-miRNA-mRNA regulatory relationship. Moreover, we explored the most remarkably enriched molecular function, cellular component, and biological processes of these DElncRNA-associated mRNA by GO and KEGG analysis. The results showed that cytokine-cytokine receptor interaction, IL-17 signaling pathway, chemokine signaling pathway, TNF signaling pathway, and Toll-like receptor (TLR) signaling pathway were enriched in the IJR group vs. the JR group. Growing evidence had revealed that the first three are involved in the development of severe lung immunopathology via recruiting B lymphocytes to the sites of pulmonary influenza virus infection and subsequently increased the susceptibility and severity in children, while TLRs as the well-known innate immune recognition receptors played the essential roles in host defense and inflammation during viral infections [32–37].

Among the pathways, the IL-17 signaling pathway presented the largest gene hits. IL-17 signaling pathway-related genes such as IL-17A, CXCL16, CCL7, IL-6, and CCL2 that regulated inflammatory responses in IJR were significantly upregulated. In addition, we found that the IL-17A gene was one of the most upregulated IL-17 signaling pathway-related genes. Omidian et al. [38] demonstrated that IL-17A was a potent proinflammatory cytokine which prevented host from pathogenic microorganism infections like IAV infection. Moreover, IL-17A was also involved in the immunopathogenesis of IAV-induced acute lung injury, which was relevant to disease severity and dysregulation of IL-17A could lead to susceptibility to infectious diseases [39]. Besides, overexpressed IL-17A could induce neutrophil activation as well as chemotaxis. When exposed to viruses, higher airway neutrophil activity increased susceptibility to viral infection [40]. Since TLR4 antagonists reduced influenza-induced mortality in rats, neutrophils could enhance susceptibility by deriving oxidized phospholipids (TLR4 agonists) and neutrophil proteases can also degrade antiviral peptides, which eliminated viral load and directly averted susceptibility to viral infection [41, 42]. Therefore, these results demonstrated that targeting lncRNAs in mediating the IL-17-dependant pathway could be a novel strategy for repressing viral infection.

**Figure 5:**
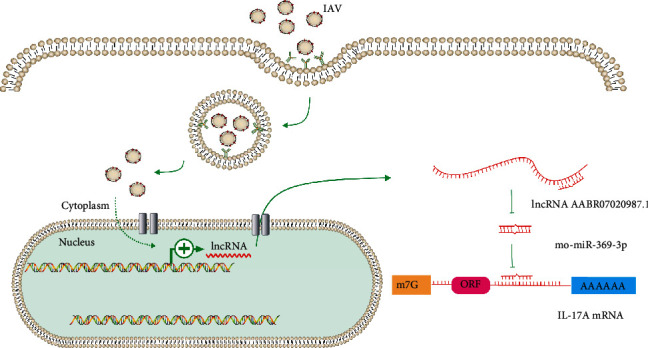
The schematic diagram of the mechanisms of the ceRNA regulatory network after influenza infection in junior rats. When the juniors were infected with influenza virus, the expression of lncRNA AABR07020987.1 expression was indirectly promoted, and then, the sponge adsorption of rno-miRNA-369-3p was enhanced, resulting in the weakened inhibition of rno-miRNA-369-3p on IL-17A mRNA.

According to bioinformatic analysis based on RNA-seq data, we speculated that lncRNA AABR07020987.1 positively affected the expression of IL-17A by acting as a ceRNA to compete with IL-17A mRNA for binding sites of rno-miR-369-3p (Figure 5). Targeting lncRNA AABR07020987.1 could be conducive to improve susceptibility to IAV in immature individuals. The lncRNA AABR07020987.1-rno-miR-369-3p-IL-17A axis could be a therapeutic potential to avert susceptibility to IAV. Therefore, this ceRNA network should be further verified by using qPCR and luciferase assays to investigate the role of IL-17A on the susceptibility to IAV in children.

## 5. Conclusion

In conclusion, 5 novel DElncRNAs (3 upregulated lncRNA (i.e., AABR07020987.1, AABR07035796.1, and Rn50_13_0829.4) and 2 downregulated (i.e., Rn50_1_0435.2 and AC141169.2)) were identified in the lung tissues of IAV-infected junior rats when compared with those in the normal junior rats and infected adult rats. Further bioinformatic analysis indicated that these DElncRNA-mediated ceRNA networks are mainly involved in the IL-17 signaling pathway and chemokine signaling pathway. Therefore, these results provided a new therapeutic strategy to improve susceptibility of influenza in children via targeting the DElncRNA-mediated ceRNA network.

## Figures and Tables

**Table 1:** Statistical result of the RNA-seq data quality test and alignment analysis.

Sample	Sequencing data				Alignment data		
	Raw reads	Clean reads	Raw bases	Clean bases	Total reads	Mapped reads	Multimap reads
AR	100132938	96394520 (96.27%)	15019940700	14459178000 (96.27%)	96394520	92470381 (95.93%)	4981812 (5.17%)
IAR	105647358	101644822 (96.21%)	15847103700	15246723300 (96.21%)	101644822	97262759 (95.69%)	5317435 (5.23%)
JR	100492130	96947954 (96.47%)	15073819500	14542193100 (96.47%)	99474110	95373552 (95.88%)	6231592 (6.26%)
IJR	102616336	99474110 (96.94%)	15392450400	14921116500 (96.94%)	96947954	92944082 (95.87%)	5626577 (5.80%)

## Data Availability

The data used to support the findings of this study are included within the article.

## Abbreviations

LncRNAs: Long noncoding RNAs
IAV: Influenza A virus
IAR: IAV-infected adult rat
AR: Adult rat
IJR: IAV-infected junior rat
JR: Normal junior rat
RNA-seq: RNA sequencing
DElncRNAs: Differently expressed lncRNAs
TCID50: Tissue culture infective dose
rRNA: Ribosomal RNA
FPKM: Fragments per Kilobase per Million Mapped Fragments
BP: Biological process
CC: Cellular component
MF: Molecular function
ANOVA: One-way analysis of variance
ceRNA: Competitive endogenous RNA
TLR: Toll-like receptor.
